# The role of ADAM17 in the T-cell response against bacterial pathogens

**DOI:** 10.1371/journal.pone.0184320

**Published:** 2017-09-06

**Authors:** Moritz Andreas Link, Karsten Lücke, Joanna Schmid, Valéa Schumacher, Thomas Eden, Stefan Rose-John, Hans-Willi Mittrücker

**Affiliations:** 1 Institute of Immunology, University Medical Center Hamburg-Eppendorf, Hamburg, Germany; 2 Institute for Biochemistry, Medical Faculty, Christian Albrechts University, Kiel, Germany; Monash University, Australia, AUSTRALIA

## Abstract

ADAM17 is a member of the A Disintegrin And Metalloproteinase family of proteases. It is ubiquitously expressed and causes the shedding of a broad spectrum of surface proteins such as adhesion molecules, cytokines and cytokine receptors. By controlled shedding of these proteins from leukocytes, ADAM17 is able to regulate immune responses. Several ADAM17 targets on T cells have been implicated in T-cell migration, differentiation and effector functions. However, the role of ADAM17 in T-cell responses is still unclear. To characterize the function of ADAM17 in T cells, we used *Adam17*^fl/fl^×*CD4cre*^+^ mice with a T-cell restricted inactivation of the *Adam17* gene. Upon stimulation, ADAM17-deficient CD4^+^ and CD8^+^ T cells were impaired in shedding of CD62L, IL-6Rα, TNF-α, TNFRI and TNFRII. Surprisingly, we could not detect profound changes in the composition of major T-cell subsets in *Adam17*^fl/fl^×*CD4cre*^+^ mice. Following infection with *Listeria monocytogenes*, *Adam17*^fl/fl^×*CD4cre*^+^ mice mounted regular listeria-specific CD4^+^ T_H1_ and CD8^+^ T-cell responses and were able to control primary and secondary infections. In conclusion, our study indicates that ADAM17 is either not required in T cells under homoeostatic conditions and for control of listeria infection or can be effectively compensated by other mechanisms.

## Introduction

Proteases of the ADAM (A Disintegrin And Metalloproteinase) family regulate various aspects of immune cell development and function. ADAM proteases cause the release of cytokines but also the ectodomain shedding of cell surface molecules including adhesion molecules and cytokine receptors [1, 2]. For ADAM17, more than 70 target proteins have been identified [2]. ADAM17 is responsible for the TNF-α release and thus for the paracrine and systemic activity of the cytokine [3, 4]. Inhibition of ADAM17 as well as global or myeloid cell-restricted deletion of ADAM17 in mice results in reduced systemic TNF-α levels following LPS treatment and protects mice from fatal endotoxemia [5, 6, 7]. Similarly, mice with defective iRhom2 (inactive Rhomboid protein 2), which facilitates trafficking of ADAM17 to the cell surface, are also less susceptible to LPS endotoxemia [8, 9]. ADAM17 has also been identified as sheddase for TNFR1 and TNFR2 [5, 10, 11, 12]. Loss of functional TNFRs reduces on one hand the sensitivity of cells towards TNF-α, on the other hand, soluble TNFRs sequester TNF-α and thereby limit its activity. Under inflammatory conditions, the IL-6Rα chain is also shed by ADAM17 [5]. In contrast to TNF-α, binding of IL-6 by soluble (s)IL-6Rα does not result in IL-6 neutralization, rather, the IL-6/sIL-6Rα can still interact with gp130 on the surface of cells and induce IL-6 signaling. Since gp130 is ubiquitously expressed, this IL-6 trans-signaling considerably widens the spectrum of IL-6 target cells [13]. Following various stimuli, L-selectin (CD62L) is rapidly shed from neutrophils and lymphocytes by ADAM17, and neutrophils deficient in ADAM17 show altered rolling and adhesion to endothelia and accelerated accumulation in the inflamed peritoneum [14, 15, 16].

Mouse T cells constitutively express *Adam17* mRNA [17] and we could recently demonstrate impaired shedding of ADAM17 targets in T cells from hypomorphic ADAM17 mice with substantially reduced ADAM17 expression or in T cells cultured with ADAM17 inhibitors [5]. ADAM17 targets are involved in diverse T-cell functions including migration and differentiation to effector cells, therefore it has been postulated that ADAM17-mediated shedding of these proteins can regulate these processes [2, 18]. Here, we use mice with a T-cell restricted ADAM17-deficiency (*Adam17*^fl/fl^×*CD4cre*^+^ mice) to investigate the role of ADAM17 in T cells under homeostatic conditions and following infection of mice with *L*. *monocytogenes*. Surprisingly, deficiency of ADAM17 in T cells did not result in substantial alterations in the composition of peripheral T cells and in the protective T-cell response to *L*. *monocytogenes*.

## Materials and methods

### Mice and *Listeria monocytogenes* infection

*Adam17*^fl/fl^ mice [6] were kindly provided by Carl Blobel and back crossed with *CD4cre*^+^ mice (B6.Cg-Tg(Cd4-cre)1Cwi/BfluJ; Jackson, Bar Harbor, ME). Due to CD4 expression in double positive thymocytes, *Adam17*^fl/fl^×*CD4cre*^+^ mice show ADAM17 deletion in peripheral CD4^+^ and CD8^+^ T cells. All mice were genotyped by PCR. ADAM17 deletion was confirmed by FACS for loss of CD62L shedding from T cells after incubation of peripheral blood cells for 30 min with PMA and ionomycin. Age- and sex-matched *Adam17*^fl/fl^×*CD4cre*^+^ and *Adam17*^fl/fl^α*CD4cre*^-^ control mice of 8 to 12 weeks of age were used in all experiments. Mice were intravenously infected with wildtype *Listeria monocytogenes* strain EGD or a *L*. *monocytogenes* strain recombinant for ovalbumin [19]. Mice received 2α10^4^ listeria in 200 μl PBS. Bacterial inocula were controlled by plating serial dilutions on PALCAM agar plates. For determination of bacterial burdens in infected mice, spleens and livers were homogenized in H_2_O, 0.5% Triton X-100 and serial dilutions of homogenates were plated on PALCAM agar. Colonies were counted after incubation at room temperature.

This study was carried out in strict accordance with the state guidelines. The protocol was approved by local ethics committee of the Behörde für Gesundheit und Verbraucherschutz of the City of Hamburg (Permit Number: 81/14). Mice were housed under specific pathogen free conditions in individually ventilated cages with standard food and water ad libitum. During infection experiments, mice were controlled daily and mice with signs of severe disease were euthanized to minimize suffering.

### Isolation and *in vitro* stimulation of cells

Cells from thymus, spleens, lymph nodes and liver were isolated by standard procedures as described before [20, 21]. For induction of shedding of surface proteins, spleen cells were incubated at 1×10^6^ cells/ml in culture medium (IMDM supplemented with 5% fetal calf serum, glutamine, pyruvate, 2-mercaptoethanol and gentamicin). Shedding was induced with 50 ng/ml phorbol 12-myristate 13-acetate (PMA, Sigma Aldrich, S. Louis, MO) and 1 μM ionomycin (Sigma Aldrich). Alternatively, cells were cultured in plates coated with anti-CD3 mAb (clone 145-2C11, Biolegend, San Diego, CA). The reaction was stopped at different time points (0, 30, 60, 120, 240 min) by placing the cell suspension on ice and adding ice cold PBS.

*In vitro* proliferation was measured by CFSE dilution assay. Spleen cells were incubated in PBS with 5μM CFSE for 15min at 37°C. Cells were washed with PBS and 4 ×10^5^ cells/well were cultured in culture medium in 96-well plates coated with anti-CD3 mAb in the presence of anti-CD28 mAb (clone 37.51, Biolegend). After three days, staining intensity of CFSE on CD4^+^ and CD8^+^ T cells was determined by flow cytometry. In parallel, cells were restimulated with 50 ng/ml PMA and 1 μM ionomycin for 4h. For the last 3.5h, 10 μg/ml brefeldin A (Sigma Aldrich) was added to the cultures to prevent cytokine secretion. Subsequently, CD40L and cytokine expression was determined by intracellular mAb staining and flow cytometry.

For the induction of cytokines, lymphocytes from spleen and liver cells were incubated at 1×10^6^ cells/ml in culture medium. Cells were stimulated for 4 h with 10^−6^ M ovalbumin peptide (OVA_257-264_; SIINFEKL) and 10^−5^ M listeriolysin O peptide (LLO_189-201_; WNEKYAQAYPNVS) (both JPT, Berlin, Germany), or with PMA and ionomycin. 10 μg/ml brefeldin A was added for the last 3.5 h of culture. Subsequently, cells were analyzed by flow cytometry [21, 22, 23].

### *In vivo* cytotoxicity assay

Spleen cells from C57BL/6 mice were incubated in culture medium with 10^-6^M of OVA_257-254_ or LCMVgp_33-41_ peptide (KAVYNFATM, JPT) at 37°C. After 1h, cells were washed with PBS and incubated in PBS with 5μM or 0.5μM CFSE for 15min at 37°C. Cells were washed with PBS and counted. CFSE^low^ and CFSE^high^ cells were mixed in a ratio of 1:1 and a total of 6×10^6^ cells was i.v. injected into naive mice or mice which had been infected with LmOVA. After 3h, spleen and liver of recipients were analyzed for CFSE-positive cells. % killing was calculated: 100 − ((% relevant peptide-pulsed cells in immunized mice / % irrelevant peptide-pulsed cells in immunized mice) / (% relevant peptide-pulsed cells in control mice/% irrelevant peptide-pulsed cells in control mice)) × 100

### Flow cytometry

For surface staining, cells were incubated for 5 min with 10 μg/ml 2.4G2 (anti-FcγRII/III; BioXCell, West Lebanon, NH) and 1:100 rat serum in PBS to minimize unspecific antibody binding. Staining was performed on ice with fluorochrome-conjugated mAb according to standard methods. Dead cells were labelled with a fixable dead cell stain (Pacific Orange succinimidyl ester; Life Technologies, Waltham, MA). For measurement of intracellular cytokines, cells were incubated with mAb against surface proteins and with Pacific Orange succinimidyl ester. After washing with PBS, cells were fixed for 20 min with PBS, 2% paraformaldehyde at room temperature. Cells were washed with PBS, 0.2% BSA, permeabilized with PBS, 0.1% BSA, 0.3% saponin (Sigma, Aldrich), and incubated in this buffer with 1% rat serum. After 5 min, fluorochrome-conjugated antibodies were added. After further 20 min, cells were washed with PBS.

Fluorochrome-labelled mAb anti-CD4 (clone RM4-5), anti-CD8α (53–6.7), anti-CD19 (eBio1D3), anti-CD25 (PC61), anti-CD44 (IM7), anti-CD62L (MEL-14), anti-IL-6Rα/CD126 (D7715A7), anti-TNFRI/CD120a (55R-286), anti-TNFRII/CD120b (TR75-89), anti-CD154/CD40L (MR1), anti-IFN-γ (XMG1.2), anti-TNF-α (MP6-XT22), anti-IL-17A (eBio17B7), and anti-FoxP3 (FJK-16s) were purchased from BD Biosciences (Heidelberg, Germany), BioLegend (San Diego, CA) or eBioscience (Frankfurt, Germany).

Cells were measured with a Canto II flow cytometer (BD Biosciences) and data were analyzed with the DIVA software (BD Bioscience) or FlowJo software (Treestar, Ashland, OR). Debris, doublets and Pacific Orange^+^ dead cells were excluded from analysis.

### Statistical analysis

Statistical analyses were performed with Prism software (GraphPad Software Inc., La Jolla, CA). Results were analyzed with the tests indicated in the figure legends. A p-value of <0.05 was considered significant (*: p<0.05; **: p<0.01; ***: p<0.001; ns: not significant).

## Results

### Impaired shedding of ADAM17 substrates by T cells from *Adam17*^fl/fl^*×CD4cre*^*+*^ mice

**Fig 1:**
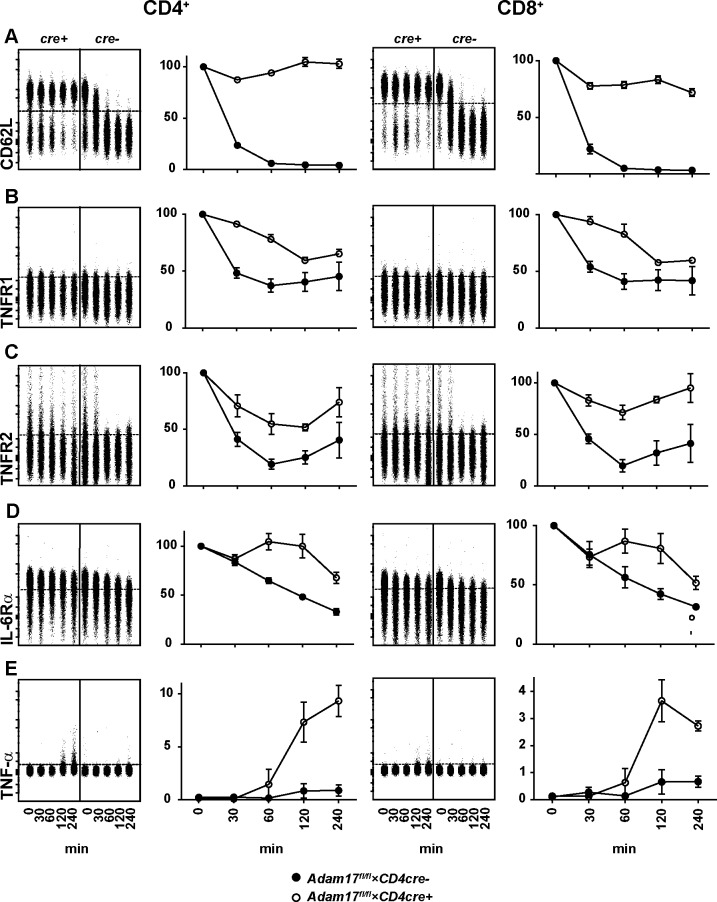
Impaired shedding of ADAM17 substrates by T cells from *Adam17*^fl/fl^×*CD4cre*^+^ mice. CD4^+^ and CD8^+^ T cells from the spleen of *Adam17*^*fl/fl*^*×CD4cre*^*-*^ control mice (*cre-*, filled symbols) and *Adam17*^*fl/fl*^*×CD4cre*^*+*^ mice (*cre+*, open symbols) were incubated for different time periods with PMA and ionomycin. Surface abundance of CD62L (A), TNFR1 (B), TNFR2 (C), IL-6Rα (D) and TNF-α (E) was analyzed by flow cytometry. Concatenated dot plots show results for CD4- and CD8-gated cells at different time points of treatment from a representative experiment. Dotted lines indicate gates as defined by isotype or unstained controls. Charts in A-D give % MFI in relation to the MFI without stimulation. Charts in E show % of surface TNF-α^+^ of CD4^+^ and CD8^+^ T cells. Charts give the mean +/-SEM of results from 6 (A) and 3 (B-E) independent experiments.

In the first set of experiments, we tested whether ADAM17-deficent T cells were able to shed verified ADAM17 substrates. Spleen cells from naive *Adam17*^fl/fl^*×CD4cre*^-^ control mice and *Adam17*^fl/fl^*×CD4cre*^*+*^ mice were incubated with PMA and ionomycin. At different time points, activation was stopped by placing the samples on ice. Cells were surface stained for CD62L, TNF-α, TNFR1, TNFR2 and IL-6Rα, and analyzed by flow cytometry (Fig 1). Following stimulation, CD4^+^ and CD8^+^ T cells from control mice rapidly lost surface expression of CD62L. In contrast, T cells from *Adam17*^fl/fl^*×CD4cre*^*+*^ mice completely failed to shed CD62L. TNF-α was not visible on the surface of T cells from control mice throughout the stimulation period. However, we detected surface TNF-α after two and four hours of stimulation on subpopulations of CD4^+^ and CD8^+^ T cells from *Adam17*^fl/fl^*×CD4cre*^*+*^ mice. TNFR1 was expressed at low levels on the surface of T cells from control mice. Comparison of MFI values revealed that control T cells rapidly lost TNFR1 surface expression following stimulation. We also observed a decrease of surface TNFR1 on CD4^+^ and CD8^+^ T cells from *Adam17*^fl/fl^*×CD4cre*^*+*^ mice, however, decrease was delayed when compared to control T cells. TNFR2 was expressed on subpopulations of CD4^+^ and CD8^+^ T cells. T cells from control mice rapidly lost this expression. In contrast, there was only marginal reduction of surface TNRF2 from ADAM17-deficient T cells. Likewise, shedding of IL-6Rα was delayed and far less pronounced from T cells of *Adam17*^fl/fl^*×CD4cre*^*+*^ mice when compared to control T cells.

**Fig 2:**
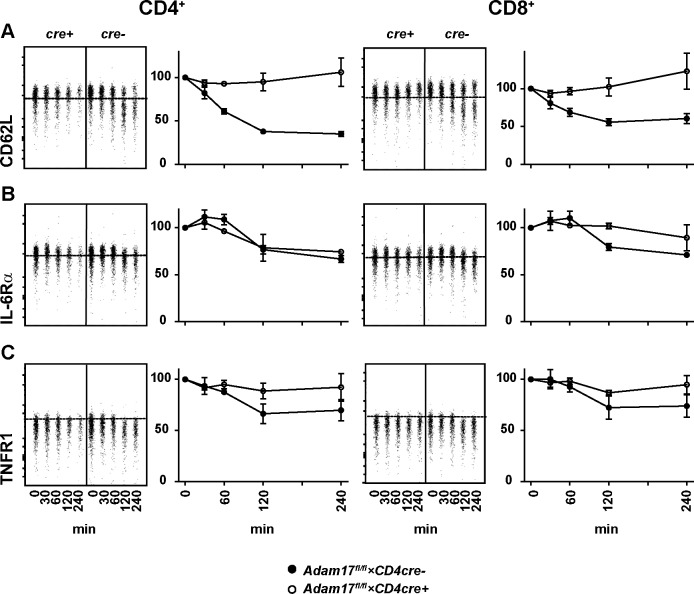
Impaired TCR-induced shedding by T cells from *Adam17*^fl/fl^×*CD4cre*^+^ mice. Spleen cells from *Adam17*^fl/fl^*×CD4cre*^*-*^ control mice (*cre-*, filled symbols) and *Adam17*^fl/fl^*×CD4cre*^*+*^ mice (*cre+*, open symbols) were incubated for different time periods in anti-CD3 mAb coated plates. Surface abundance of CD62L (A), IL-6Rα (B) and TNFR1 (C) was analyzed by flow cytometry. Concatenated dot plots show results for CD4- and CD8-gated cells at different time points of treatment from a representative experiment. Dotted lines in the diagrams indicate gates as defined by isotype or unstained controls. Charts in A-C give % MFI in relation to the MFI without stimulation. Charts give the mean +/- SEM of results from 2 independent experiments.

Spleen cells were also incubated in plates coated with anti-CD3 mAb and surface expression of CD62L, IL-6Rα and TNFR1 was determined (Fig 2). In comparison to PMA and ionomycin treatment, shedding of these proteins was less pronounced and delayed after anti-CD3 mAb treatment. However, we also observed impaired shedding of CD62L, IL-6Rα and TNFR1 from T cells from *Adam17*^fl/fl^*×CD4cre*^*+*^ mice when compared to T cells from control mice.

In conclusion, deficiency of ADAM17 completely prevented shedding of CD62L indicating the efficient deletion of *Adam17* in CD4^+^ and CD8^+^ T cells in *Adam17*^fl/fl^*×CD4cre*^*+*^ mice. This result also suggests that reduction of other ADAM17 substrates from the surface of activated T cells from *Adam17*^fl/fl^*×CD4cre*^*+*^ mice was most likely due to ADAM17-independent mechanisms, such as shedding by other proteases or altered surface turnover.

### T-cell development and peripheral T-cell composition in *Adam17*^fl/fl^*×CD4cre*^*+*^ mice

**Fig 3:**
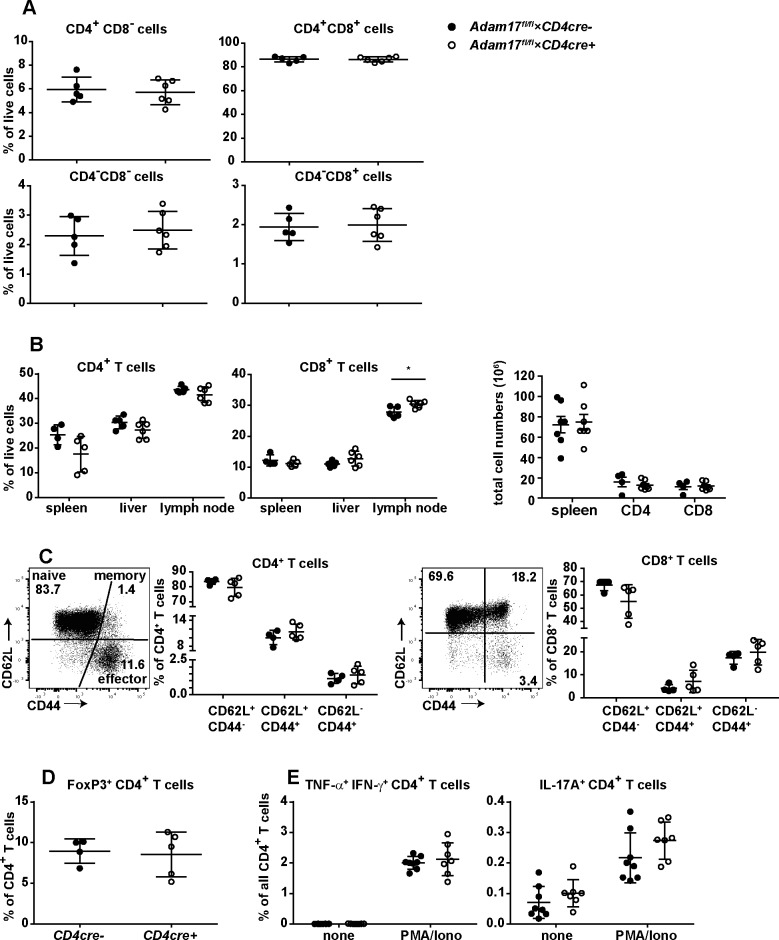
Regular T-cell composition in *Adam17*^fl/fl^×*CD4cre*^+^ mice. (A) Frequencies of CD4^-^CD8^-^, CD4^+^CD8^+^, CD4^+^CD8^-^ and CD4^-^CD8^+^ thymocytes in *Adam17*^fl/fl^×*CD4cre*^-^ and *Adam17*^fl/fl^×*CD4cre*^+^ mice. (B) Frequencies of CD4^+^ and CD8^+^ T cells in spleen, liver and inguinal lymph nodes, as well as numbers of CD4^+^ and CD8^+^ T cells in the spleen. (C) Distribution of CD62L^+^CD44^-^ (naive), CD62L^-^CD44^+^ (effector/effector memory) and CD62L^+^ CD44^+^ T cells (memory) in spleen. Dot plots display representative CD44 and CD62L staining profiles of CD4- or CD8-gated cells. (D) Frequencies of FoxP3^+^ CD4^+^ T_reg_ cells. (E) Frequencies of TNF-α^+^ IFN-γ^+^ CD40L^+^ T_H1_ cells and IL-17A^+^ CD40L^+^ T_H17_ cells. Cytokines and CD40L expression were determined by intracellular mAb staining following stimulation of spleen cells for 4 h with PMA (50 ng/ml) and ionomycin (1 μM). Scatter plots give results for individual mice and the means +/- SD from representative experiments. Experiments were repeated at least 2 times. Groups were compared with student’s t test. A p-value of <0.05 was considered significant.

Due to the impaired shedding of proteins involved in T-cell function, we wondered whether ADAM17-deficiency causes alterations in maturation and composition of peripheral CD4^+^ and CD8^+^ T cells. Therefore, the cellular composition of thymus, spleen, inguinal lymph nodes and liver of *Adam17*^fl/fl^*×CD4cre*^*+*^ mice was analyzed by flow cytometry. We observed a similar distribution of CD4^-^CD8^-^ (double negative), CD4^+^CD8^+^ (double positive), and CD4^+^CD8^-^ and CD4^-^CD8^+^ (single positive) cells in the thymus of *Adam17*^fl/fl^*×CD4cre*^*+*^ and *Adam17*^fl/fl^*×CD4cre*^*-*^ control mice (Fig 3A, S1 Fig). Likewise, *Adam17*^fl/fl^*×CD4cre*^*+*^ mice showed a regular composition of conventional CD4^+^ and CD8^+^ T cells in spleen, inguinal lymph nodes and liver as well as similar total numbers of CD4^+^ and CD8^+^ T cells in the spleen (Fig 3B). In some experiments, we observed slightly reduced CD4^+^ and increased CD8^+^ T-cell frequencies, however, these alterations were not consistent in all experiments. T cells from spleen, lymph nodes and liver were stained for CD44 and CD62L to determine the distribution of naive (CD44^-^CD62L^+^), effector/effector memory (CD44^+^CD62L^-^) and central memory T cells (CD44^+^CD62L^+^). There was a similar distribution of these T-cell subpopulations in *Adam17*^fl/fl^*×CD4cre*^*+*^ and *Adam17*^fl/fl^*×CD4cre*^*-*^ control mice (Fig 3C).

IL-6 plays a fundamental role in the differentiation of T_H17_ cells and prevents the formation of peripheral T_reg_ cells. Since impaired shedding of IL-6Rα might influence these differentiation pathways, we determined the frequencies of FoxP3^+^ CD4^+^ T_reg_ cells (Fig 3D) as well as T_H17_ cells and T_H1_ cells (Fig 3E). T_reg_ cells were detected by intracellular FoxP3 staining (S1 Fig). For determination of T_H1_ and T_H17_ cells, spleen cells were incubated with PMA/ionomycin or without stimulation. After 4h, intracellular expression of CD40L, which is upregulated on all conventional CD4^+^ T_H_ cells following TCR stimulation, as well as IFN-γ, TNF-α and IL-17A was determined by flow cytometry. We observed similar frequencies of T_reg_ cells, and after stimulation equal frequencies of CD40L^+^ IFN-γ^+^ TNF-α^+^ T_H1_ cells and CD40L^+^ IL-17A^+^ T_H17_ cells among CD4^+^ T cells from spleens of *Adam17*^fl/fl^*×CD4cre*^*+*^ and *Adam17*^fl/fl^*×CD4cre*^*-*^ mice.

In conclusion, deficiency of ADAM17 in CD4^+^ and CD8^+^ T cells did not cause profound changes in the composition, distribution and differentiation status of conventional T cells.

### *In vitro* responses of T cells from *Adam17*^fl/fl^*×CD4cre*^*+*^ mice

In the next set of experiments, we tested the function of T cells *in vitro*. Spleen cells from *Adam17*^fl/fl^*×CD4cre*^*+*^ and *Adam17*^fl/fl^*×CD4cre*^*-*^ mice were labelled with CFSE and cultured with anti-CD3 mAb and anti-CD28 mAb. After 3 days, proliferation was determined by loss of CFSE staining (S2 Fig). We detected similar CFSE staining in CD4^+^ T cells from both mouse strains but somewhat higher CFSE staining in CD8^+^ T cells from *Adam17*^fl/fl^*×CD4cre*^*-*^ control mice, indicating slightly less extensive proliferation of these cells. After 3 days, T cells were, in addition, re-stimulated for 4h with PMA and ionomycin and cytokine expression was determined by intracellular staining and flow cytometry (S2 Fig). The vast majority of CD4^+^ T cells from both mouse strains responded with upregulation of CD40L. A larger fraction of ADAM17-deficient CD4^+^ T cells were able to produce TNF-α and IFN-γ. Stimulation resulted in induction of TNF-α and IFN-γ in most CD8^+^ T cells from both mouse strains. Here, we detected slightly lower frequencies of TNF-α^+^ CD8^+^ T cells in cultures of *Adam17*^fl/fl^*×CD4cre*^*+*^ spleen cells.

In summary, ADAM17 deficiency did not substantially alter the *in vitro* T-cell response to polyclonal stimulation. In some assays, ADAM17-deficient T cells showed stronger responses, however, differences were in all cases only very modest.

### T-cell responses to *Listeria monocytogenes* infection in *Adam17*^fl/fl^*×CD4cre*^*+*^ mice

To test the role of ADAM17 in the generation and function of a T-cell response *in vivo*, we applied the *Listeria monocytogenes* infection model. *L*. *monocytogenes* infection induces strong CD4^+^ T_H1_ and CD8^+^ T-cell responses, which are essential for the clearance of the bacteria. Furthermore, control of *L*. *monocytogenes* highly depends on TNF-α [24, 25, 26, 27].

Mice were i.v. infected with 2×10^4^ bacteria of a *L*. *monocytogenes* strain recombinant for ovalbumin (LmOVA) [19] and the T-cell response in spleen and liver was analyzed at different time points post-infection. For C57BL/6 mice, immunodominant CD8^+^ T-cell epitopes from *L*. *monocytogenes* are currently not known. Since ovalbumin contains a strong CD8^+^ T-cell epitope (OVA_257-264_), application of LmOVA allows determination of ovalbumin-specific CD8^+^ T-cell responses generated during *L*. *monocytogenes* infection of C57BL/6 mice.

**Fig 4:**
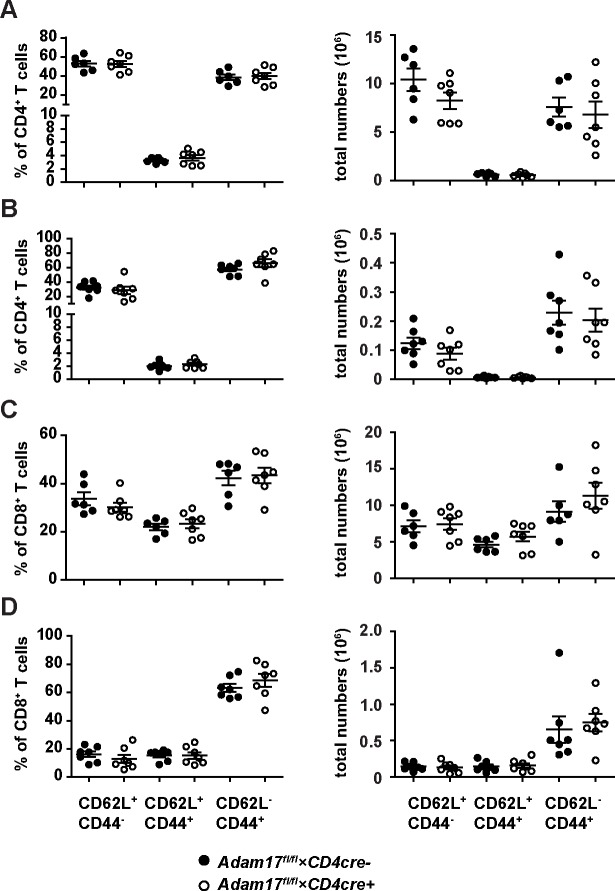
T-cell composition in *Adam17*^fl/fl^×*CD4cre*^+^ mice infected with *Listeria monocytogenes*. *Adam17*^fl/fl^×*CD4cre*^-^ and *Adam17*^fl/fl^×*CD4cre*^+^ mice were infected i.v. with 2×10^4^ LmOVA. After 8 days, frequencies and numbers of CD62L^+^CD44^-^ (naive), CD62L^-^CD44^+^ (effector/effector memory) and CD62L^+^ CD44^+^ (memory) CD4^+^ (A, B) and CD8^+^ T cells (C, D) in spleen (A, C) and liver (B, D) was determined. Scatter plots give results for individual mice and the means +/- SD from a representative experiment. Experiments were repeated 2 times. Groups were compared with student’s t test. A p-value of <0.05 was considered significant.

Compared to naive mice (Fig 3), we observed a shift in frequencies and numbers from CD44^-^CD62L^+^ naive to CD44^+^CD62L^-^ effector CD4^+^ and CD8^+^ T cells in spleen (Fig 4A and 4C) and liver (Fig 4B and 4D) at 8 days post *L*. *monocytogenes* infection. However, changes in T-cell compositions were similar in *Adam17*^fl/fl^*×CD4cre*^*+*^ and *Adam17*^fl/fl^*×CD4cre*^*-*^ mice. We also determined the expression levels of CD62L and IL-6Rα on activated CD44^+^ CD4^+^ and CD44^+^ CD8^+^ T cells (S3 Fig). Surface expression of both proteins was reduced on CD44^+^ T cells following infection. However, T cells from *Adam17*^fl/fl^*×CD4cre*^*+*^ and *Adam17*^fl/fl^*×CD4cre*^*-*^ mice did not differ in CD62L and IL-6Rα expression levels.

**Fig 5:**
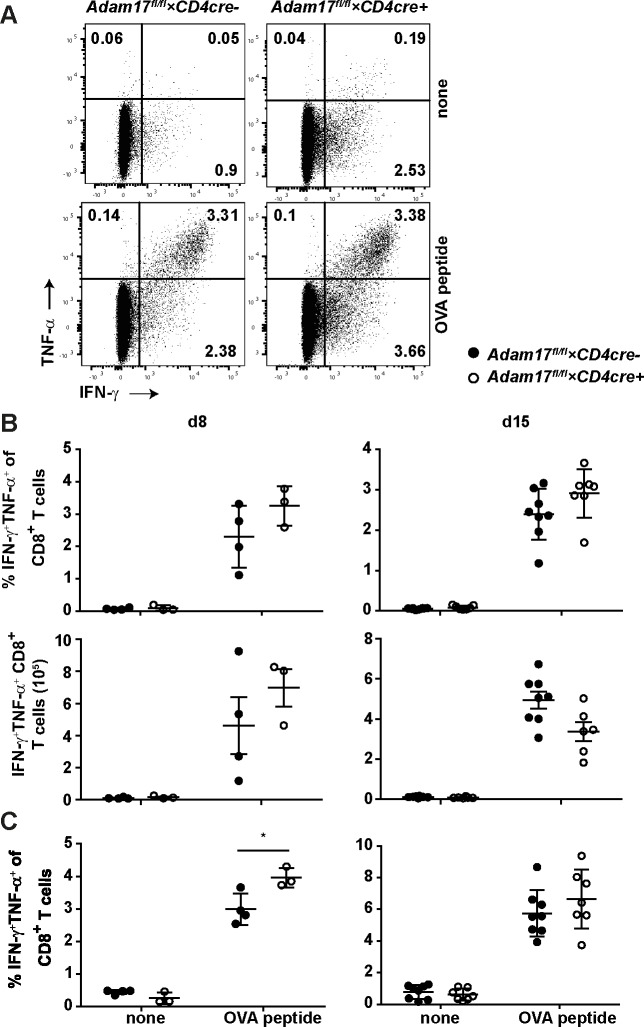
Listeria-specific CD8^+^ T-cell response in *Adam17*^fl/fl^×*CD4cre*^+^ mice. *Adam17*^fl/fl^×*CD4cre*^-^ and *Adam17*^fl/fl^×*CD4cre*^+^ mice were infected with i.v. 2×10^4^ LmOVA. After 8 and 15 days of infection, lymphocytes isolated from spleen and liver were incubated for 4 h without (none) or with OVA_257-264_ peptide. Subsequently, the frequency of TNF-α^+^ IFN-γ^+^ CD8^+^ T cells was analyzed by flow cytometry. (A) Dot plots show representative results for IFN-γ and TNF-α expression in CD8-gated cells from the spleen at d8 post-infection. (B) Scatter plots give the frequencies and numbers of TNF-α^+^ IFN-γ^+^ CD8^+^ T cells from spleens of individual mice and the means +/- SD from representative experiments at days 8 (left) and 15 (right) post-infection. (C) Scatter plots give the frequencies of TNF-α^+^ IFN-γ^+^ CD8^+^ T cells from livers of individual mice and the means +/- SD from representative experiments. Experiments were repeated 2 times. Groups were compared with student’s t test. A p-value of <0.05 was considered significant.

To determine the formation of specific CD8^+^ T cells, cells isolated from spleen and liver were incubated with OVA_257-264_ peptide and the production of IFN-γ and TNF-α were measured by intracellular cytokine staining and flow cytometry. At day 8 and 15 post infection, we observed high frequencies and numbers of OVA_257-264_-specific CD8^+^ T cells in spleens, however, frequencies and numbers were similar in *Adam17*^fl/fl^*×CD4cre*^*+*^ and *Adam17*^fl/fl^*×CD4cre*^*-*^ mice (Fig 5A and 5B). In some experiments, we observed slightly higher frequencies of cytokine producing CD8^+^ T cells in the liver of *Adam17*^fl/fl^*×CD4cre*^*+*^ mice, however, these differences were not consistent in all experiments (Fig 5C).

**Fig 6:**
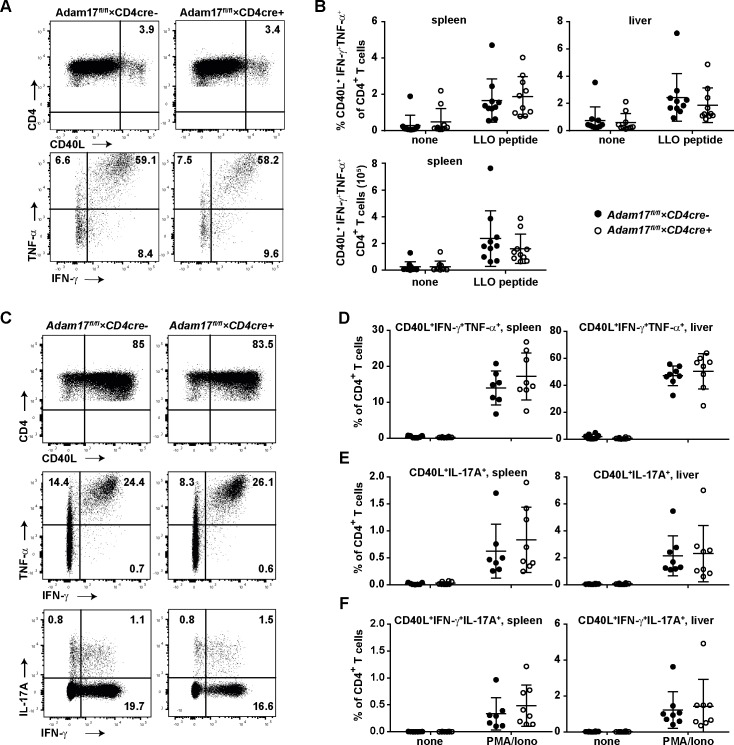
CD4^+^ T_H1_-cell response in *Adam17*^fl/fl^×*CD4cre*^+^ mice. *Adam17*^fl/fl^×*CD4cre*^-^ and *Adam17*^fl/fl^×*CD4cre*^+^ mice were infected i.v. with 2×10^4^ LmOVA. (A, B) After 8 days of infection, lymphocytes isolated from spleen and liver were incubated for 4 h without (none) and with LLO_189-201_ peptide. Frequencies of CD40L^+^ TNF-α^+^ IFN-γ^+^ CD4^+^ T cells were measured by flow cytometry. (A) Representative dot plots for spleen cells stimulated with LLO_189-201_ peptide. Upper panel: % of CD40L^+^ cells in CD4-gated cells. Lower panel: % of TNF-α^+^ and IFN-γ^+^ cells among CD4^+^ CD40L^+^ cells. (B) Scatter plots give the results for CD40L^+^ TNF-α^+^ IFN-γ^+^ CD4^+^ T_H1_ cells results for individual mice and the means +/- SD from a representative experiment. Experiments were repeated 2 times. (C-F) After 15 days of infection, lymphocytes isolated from spleen and liver were incubated for 4 h without (none) and with PMA/ionomycin. Subsequently, the frequencies of T_H1_ (CD40L^+^ TNF-α^+^ IFN-γ^+^), T_H17_ (CD40L^+^ IL-17A^+^) and T_H1_/T_H17_ (CD40L^+^ IL-17A^+^ IFN-γ^+^) cells were determined by flow cytometry. (C) Representative dot plots for spleen cells stimulated with PMA/ionomycin. Upper panel: % of CD40L^+^ cells in CD4-gated cells. Middle panel: % of TNF-α^+^ and IFN-γ^+^ cells among CD4^+^ CD40L^+^ cells. Lower panel: % of Il-17A^+^ and IFN-γ^+^ cells among CD4^+^ CD40L^+^ cells. Scatter plots give frequencies of CD40L^+^ TNF-α^+^ IFN-γ^+^ (D), CD40L^+^ IL-17A^+^ (E) and CD40L^+^ IL-17A^+^ IFN-γ^+^ (F) in spleen and liver. Results for individual mice and the means+/-SD from a representative experiment are shown. Experiments were repeated 2 times. Groups were compared with student’s t test. A p-value of <0.05 was considered significant.

Likewise, following stimulation with the peptide listeriolysin O peptide (LLO_189-201_) which is immunodominant for CD4^+^ T cells in C57BL/6 mice, similar frequencies of IFN-γ^+^TNF-α^+^CD40L^+^ CD4^+^ T_H1_ cells were detected in spleens and livers of infected *Adam17*^fl/fl^*×CD4cre*^*+*^ and *Adam17*^fl/fl^*×CD4cre*^*-*^ mice (Fig 6A and 6B).

Cells from listeria-infected mice *Adam17*^fl/fl^*×CD4cre*^*+*^ were also polyclonally stimulated with PMA/ionomycin to test whether ADAM17-deficiency resulted in a general shift in the cytokine response of CD4^+^ T cells (Fig 6C–6F). Again, we detected comparable frequencies of IFN-γ^+^ TNF-α^+^ CD40L^+^ T_H1_ cells (Fig 6D) and of IL-17A^+^CD40L^+^ T_H17_ cells (Fig 6E), as well as a of CD4^+^ T cells with a mixed IFN-γ^+^ IL-17A^+^ CD40L^+^ T_H1_/T_H17_-cell phenotype (Fig 6F) in *Adam17*^fl/fl^*×CD4cre*^*+*^ and *Adam17*^fl/fl^*×CD4cre*^*-*^ mice.

**Fig 7:**
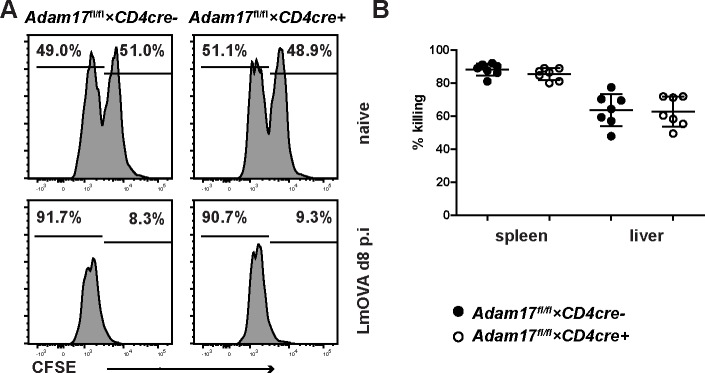
Cytotoxic activity of CD8^+^ T cells in infected *Adam17*^fl/fl^×*CD4cre*^+^ mice. *Adam17*^fl/fl^×*CD4cre*^-^ and *Adam17*^fl/fl^×*CD4cre*^+^ mice were infected i.v. with 2×10^4^ LmOVA. After 8 days, mice were injected with a 1:1 mixture of spleen cells labelled with different levels of CFSE and loaded with OVA_257-264_ or the irrelevant LCMVgp_33-41_ peptide. Three hours later, the ratio of CFSE^high^ LLO_189-201_-loaded cells to CFSE^low^ LCMVgp_33-41_-loaded cells was determined in spleen and liver. (A) Representative histograms for CFSE-positive cells recovered from naive (upper panel) and infected mice (lower panel). (B) Scatter plots gives % killing of LLO_189-201_-loaded cells in spleen and liver for individual mice and the means +/- SD from a representative experiment. The experiment was performed twice with similar outcome. Groups were compared with student’s t test. A p-value of <0.05 was considered significant.

Finally, the cytolytic activity of CD8^+^ T cells was tested (Fig 7). *Adam17*^fl/fl^*×CD4cre*^*+*^ and *Adam17*^fl/fl^*×CD4cre*^*-*^ mice were infected with LmOVA. Eight days later, mice received a 1:1 mixture of spleen cells loaded either with OVA_257-264_ or with an irrelevant peptide. After further 3h, frequencies of both peptide-loaded cell populations were determined in spleen and liver. Compared to non-infected recipients, we observed a strong reduction of OVA_257-264_-loaded spleen cells in spleens and livers of infected mice, indicating that LmOVA infection had induced OVA-specific cytotoxic CD8^+^ T cells. However, we did not observe a difference in the cytotoxic activity between *Adam17*^fl/fl^*×CD4cre*^*+*^ and *Adam17*^fl/fl^*×CD4cre*^*-*^ mice.

In conclusion, ADAM17 deficiency in CD4^+^ and CD8^+^ T cells did not substantially alter the T-cell response to *L*. *monocytogenes* in terms of frequencies of responding cells, expression profile of the analyzed cytokines and cytotoxicity.

### Control of *Listeria monocytogenes* infection in *Adam17*^fl/fl^*×CD4cre*^*+*^ mice

**Fig 8:**
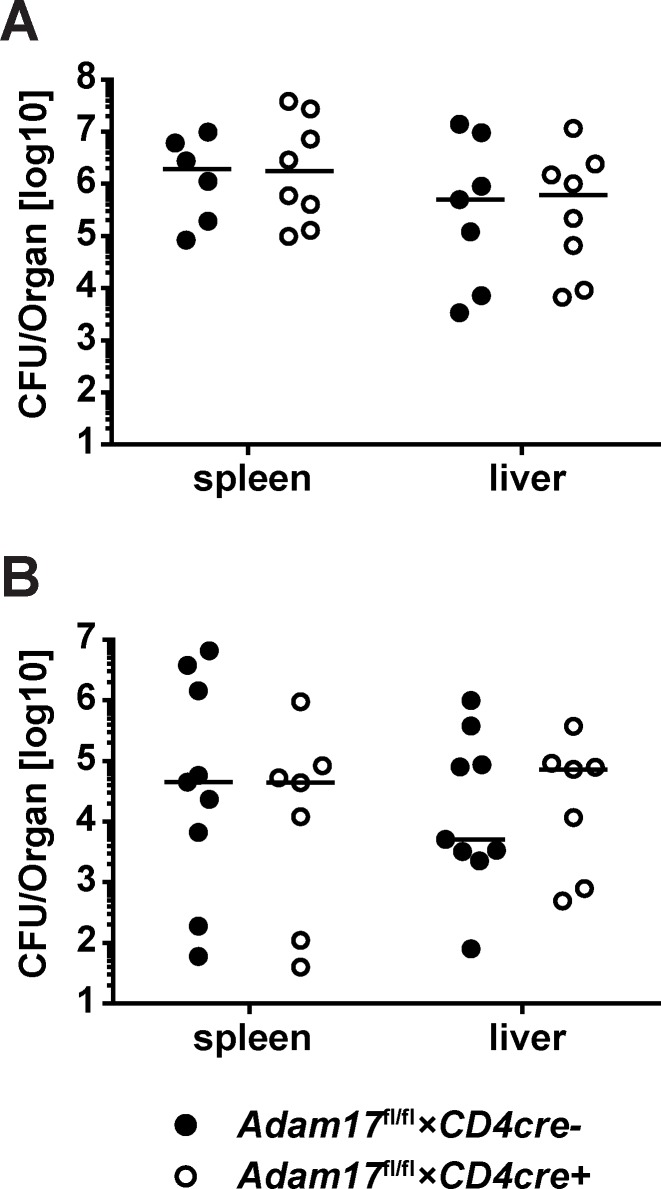
Control of primary and secondary *Listeria monocytogenes* infection in *Adam17*^fl/fl^×*CD4cre*^+^ mice. (A) *Adam17*^fl/fl^×*CD4cre*^-^ and *Adam17*^fl/fl^×*CD4cre*^+^ mice were infected i.v. with 2×10^4^ LmOVA. Five days post-infection, listeria titers in spleen and liver were determined. (B) Mice were i.p. infected with 2×10^3^ wildtype listeria. After 40 days, animals were i.v. re-infected with 1×10^5^ wildtype listeria. Two days post-infection, listeria titers in spleen and liver were determined. Scatter plots give results for individual mice and the median for a representative experiment. Experiments were repeated 2 times. Groups were compared with the Mann-Whitney U test. A p-value of <0.05 was considered significant.

TNF-α is fundamental for the immune response to *L*. *monocytogenes*. Thus, impaired shedding of TNF-α, TNFR1 and TNFR2 from T cells could result in impaired control of listeria. *Adam17*^fl/fl^*×CD4cre*^*+*^ and *Adam17*^fl/fl^*×CD4cre*^*-*^ mice were i.v. infected with 2×10^4^ listeria and 5 days later, listeria titers in spleens and livers were determined (Fig 8A). There was no significant difference in the bacterial titers of spleen and liver between *Adam17*^fl/fl^*×CD4cre*^*+*^ and *Adam17*^fl/fl^*×CD4cre*^*-*^ mice, indicating that shedding of TNF-α or of other surface proteins from T cells is not required for the control of *L*. *monocytogenes*.

Control of secondary *L*. *monocytogenes* infection strongly relies on the acquired T-cell response and might therefore be affected in *Adam17*^fl/fl^*×CD4cre*^*+*^ mice. To test the efficacy of these T-cells, *Adam17*^fl/fl^*×CD4cre*^*-*^ and *Adam17*^fl/fl^*×CD4cre*^*+*^ mice were infected with *L*. *monocytogenes* and after 40 days reinfected with a lethal listeria dose. Two days later, titers in spleen and liver were determined (Fig 8B). None of the reinfected mice succumbed to the high dose infection and *Adam17*^fl/fl^*×CD4cre*^*+*^ and *Adam17*^fl/fl^*×CD4cre*^-^ mice harbored comparable *L*. *monocytogenes* titers in spleen and liver. This result implies that *Adam17*^fl/fl^*×CD4cre*^*+*^ mice can generate listeria-specific memory T cells which are effective in protection upon secondary listeria infection. In conclusion, *Adam17*^fl/fl^*×CD4cre*^*+*^ were not impaired in their control of primary and secondary *L*. *monocytogenes* infection.

## Discussion

ADAM17 is constitutively expressed in T cells and expression only marginally changes following T-cell activation [17]. Functional characterization of ADAM17-deficient T cells *in vitro* showed altered shedding of surface proteins such as CD62L, TNF-α, TNFR1, TNFR2 and IL-6Rα, all considered relevant for CD4^+^ and CD8^+^ T-cell function, as well as modest changes in proliferation and cytokine production. Surprisingly, ADAM17-deficiency apparently did not influence the T-cell function *in vivo*. We did not observe significant changes in maturation, migration, differentiation patterns and function of CD4^+^ and CD8^+^ T cells in naive and listeria-infected mice. The composition of conventional T-cell subsets was unchanged, there was no shift in T_reg_ or T_H17_-cell differentiation, and mice were able to mount an effective CD8^+^ and T_H1_ response to *L*. *monocytogenes*. We detected similar listeria titers in spleen and liver of *Adam17*^fl/fl^*×CD4cre*^*+*^ and *Adam17*^fl/fl^*×CD4cre*^-^ mice at day 5 of primary and day 2 of secondary infection indicating that ADAM17-deficient T cells were not impaired in controlling *L*. *monocytogenes*. During this study, we infected a total of 96 *Adam17*^fl/fl^*×CD4cre*^-^ and 97 *Adam17*^fl/fl^*×CD4cre*^+^ mice and followed these mice for up to 40 days. We lost 3 *Adam17*^fl/fl^*×CD4cre*^-^ mice (3.1%) and 8 *Adam17*^fl/fl^*×CD4cre*^+^ mice (8.2%) due to infection, which further confirms that ADAM17-deficiency does not substantially weakens the T-cell response to *L*. *monocytogenes*.

Naive and memory T cells require the addressin CD62L for entering the T-cell zone of secondary lymphoid tissues at high endothelial venules. Subsequent activation in the T-cell zone causes rapid loss of CD62L surface expression. Our *in vitro* experiments demonstrate that CD62L shedding from T cells is strictly dependent on ADAM17. However, we did not observe substantial changes in T-cell development and distribution as well as in the T-cell response to *L*. *monocytogenes*. Our results confirm studies demonstrating normal homeostatic T-cell distribution in mice with a protease resistant CD62L protein [28, 29]. Thus, rapid CD62L shedding from activated T cells appears to be not essential for their function. mRNA expression analyses indicate that activated CD8^+^ T cells downregulate *Sell* mRNA (coding for CD62L) during listeria infection [17], which might compensate for defective shedding.

We observed impaired shedding of TNF-α and TNFRI, and delayed shedding of TNFRII from T cells of *Adam17*^fl/fl^*×CD4cre*^*+*^ mice. These defects did neither interfere with T-cell differentiation under homeostatic conditions nor with formation of a T-cell response against *L*. *monocytogenes*. Shedding of TNF-α from T cells was also not required for listeria control during primary and secondary infection. In the case of TNFRI and TNFRII other protease might compensate for the ADAM17-deficiency in T cells. Several studies could demonstrate that membrane-bound TNF-α is active [30, 31, 32, 33, 34] and capable of providing partial protection against *L*. *monocytogenes* infection [27, 31, 33]. In addition, protection against listeria mainly relies on TNF-α produced by myeloid cells and only under conditions with high titers, T-cell derived TNF-α becomes important [26]. Finally, control of secondary listeria infection does not require TNF-α [26, 35]. Together, these results could well explain why a defect in shedding of TNF-α in *Adam17*^fl/fl^*×CD4cre*^*+*^ mice does not substantially impair the control of *L*. *monocytogenes*.

IL-6 supports the formation of T_H17_ cells and suppresses the induction of peripheral T_reg_ cells [36, 37, 38, 39]. Furthermore, there is controversial data on the role of IL-6 in T_H1_ responses [40, 41]. Based on these studies, one could postulate that IL-6Rα shedding restricts the response of activated CD4^+^ T cells to IL-6 and thereby regulates CD4^+^ T-cell differentiation. In our *in vitro* study, we observe impaired IL-6Rα shedding from T cells of *Adam17*^fl/fl^*×CD4cre*^*+*^ mice which is consistent with our results from experiments with T cells of hypomorphic ADAM17 mice or with metalloprotease inhibitors [5]. However, ADAM17-deficiency affected neither the composition of T-cell subsets under homeostatic conditions nor the formation of the T-cell response against *L*. *monocytogenes*. Listeria infection of mice is considered to be a T_H1_ model and thus provides only very limited information on T_H17_-cell differentiation. In addition, it could recently be shown that T_H17_ differentiation requires IL-6 presentation by IL-6Rα^+^ dendritic cells and is largely independent from IL-6Rα expression on T cells [38]. However, *Adam17*^fl/fl^*×CD4cre*^*+*^ mice also showed normal frequencies of T_reg_ cells and generated a regular T_H1_ and CD8^+^ T-cell response to *L*. *monocytogenes*. Thus, ADAM17-mediated shedding of IL-6Rα is either not important for these processes or compensated by other mechanisms such as shedding by other proteases, receptor internalization or down-regulation of *Il6Ra* mRNA expression in activated T cells [5, 17].

In conclusion, our study indicates that although T cells bear several potential ADAM17 targets on their surface, ADAM17-deficiency does not result in profound changes of peripheral T-cell composition under homeostatic conditions and does not impair the T_H1_ and CD8^+^ T-cell response against *L*. *monocytogenes*. ADAM17-mediated shedding might simply be not required in these processes. Alternatively, ADAM17 targets could be shed by other proteases, removed from the cell surface by internalization or downregulated on the mRNA expression level. Our analysis was restricted to the T-cell response against *L*. *monocytogenes*. However, it is well conceivable that under different circumstances, such as responses to other pathogens or to tumors or in autoimmunity, T cells might require ADAM17 for proper function.

## Supporting information

S1 Fig T-cell composition in *Adam17*^fl/fl^×*CD4cre*^+^ mice. (A) Representative dot plot to define double negative, double positive and single positive thymocytes. Numbers give % of cells within regions. (B) Representative CD25 and Foxp3 staining of CD4-gated cells.
(PDF)

S2 Fig *In vitro* response of T cells from *Adam17*^fl/fl^×*CD4cre*^+^ mice. Spleen cells from *Adam17*^fl/fl^×*CD4cre*^-^ and *Adam17*^fl/fl^×*CD4cre*^+^ mice were labelled with CFSE and stimulated with anti-CD3 mAb and anti-CD28 mAb. After 3 days, CFSE expression was determined on CD4^+^ and CD8^+^ T cells. At this time point, T cells were also re-stimulated for further 4h with PMA and ionomycin or were left without stimulation (none). Subsequently, expression of CD40L, TNF-α and IFN-γ was determined by intracellular staining and flow cytometry. (A) Representative histograms for CFSE expression of CD4^+^ and CD8^+^ T cells from *Adam17*^fl/fl^×*CD4cre*^-^ (light grey) and *Adam17*^fl/fl^×*CD4cre*^+^ mice (dark grey). Charts give the MFI for individually analyzed samples. (B) Frequencies of CD40L^+^, CD40L^+^TNF-α^+^ and CD40L^+^IFN-γ^+^ CD4^+^ T cells (left), as well as of TNF-α^+^ and IFN-γ^+^ CD8^+^ T cells (right). Charts give the mean+/-SD of triplicate cultures for each mouse strain. Groups were compared with student’s t test. A p-value of <0.05 was considered significant. Data are representative for two independent experiments with similar outcome.
(PDF)

S3 Fig CD62L and IL-6Rα surface expression on CD4^+^ and CD8^+^ T cells from *Adam17*^fl/fl^×*CD4cre*^+^ mice. *Adam17*^fl/fl^×*CD4cre*^-^ and *Adam17*^fl/fl^×*CD4cre*^+^ mice were infected with 2×10^4^ LmOVA. Spleen cells from naive mice (A) and mice infected for 8 (B) and 15 days (C) were analyzed for surface expression by flow cytometry. Scatter plots give MFI (mean fluorescence intensity) for CD62L and IL-6Rα on CD44^+^CD4^+^ and CD44^+^CD8^+^ T cells. Results for individually analyzed mice and mean +/- SD are presented. Experiments were repeated 2 times.
(PDF)
