# Supplementary material for: The role of ADAM17 in the T-cell response against bacterial pathogens
Source: PLoS One. 2017 Sep 6;12(9):e0184320. doi: 10.1371/journal.pone.0184320 (PMC5587322; doi:10.1371/journal.pone.0184320)
Supplement: S1 Fig — (A) Representative dot plot to define double negative, double positive and single positive thymocytes. Numbers give % of cells within regions. (B) Representative CD25 and Foxp3 staining of CD4-gated cells. (PDF) [file pone.0184320.s001.pdf]

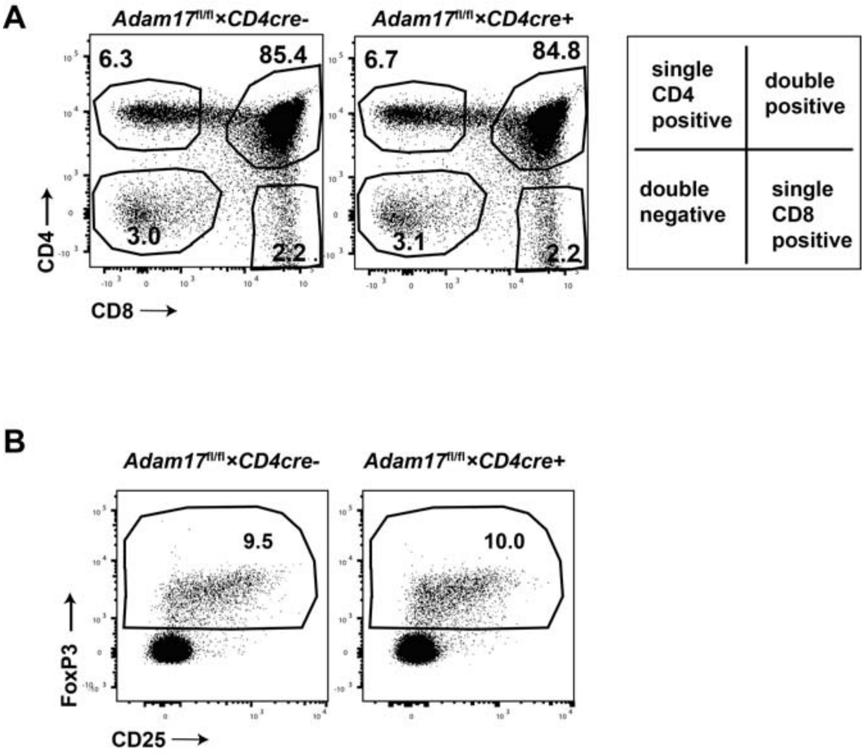

S1 Fig. T-cell composition in *Adam17*$^{\mathbf{fl/fl}}$×*CD4cre*$^{+}$ mice.

(A) Representative dot plot to define double negative, double positive and single positive thymocytes. Numbers give % of cells within regions. (B) Representative CD25 and Foxp3 staining of CD4-gated cells.
