# Supplementary material for: The role of ADAM17 in the T-cell response against bacterial pathogens
Source: PLoS One. 2017 Sep 6;12(9):e0184320. doi: 10.1371/journal.pone.0184320 (PMC5587322; doi:10.1371/journal.pone.0184320)
Supplement: S2 Fig — Spleen cells from Adam17fl/fl×CD4cre- and Adam17fl/fl×CD4cre+ mice were labelled with CFSE and stimulated with anti-CD3 mAb and anti-CD28 mAb. After 3 days, CFSE expression was determined on CD4+ and CD8+ T cells. At this time point, T cells were also re-stimulated for further 4h with PMA and ionomycin or were left without stimulation (none). Subsequently, expression of CD40L, TNF-α and IFN-γ was determined by intracellular staining and flow cytometry. (A) Representative histograms for CFSE expression of CD4+ and CD8+ T cells from Adam17fl/fl×CD4cre- (light grey) and Adam17fl/fl×CD4cre+ mice (dark grey). Charts give the MFI for individually analyzed samples. (B) Frequencies of CD40L+, CD40L+TNF-α+ and CD40L+IFN-γ+ CD4+ T cells (left), as well as of TNF-α+ and IFN-γ+ CD8+ T cells (right). Charts give the mean+/-SD of triplicate cultures for each mouse strain. Groups were compared with student’s t test. A p-value of <0.05 was considered significant. Data are representative for two independent experiments with similar outcome. (PDF) [file pone.0184320.s002.pdf]

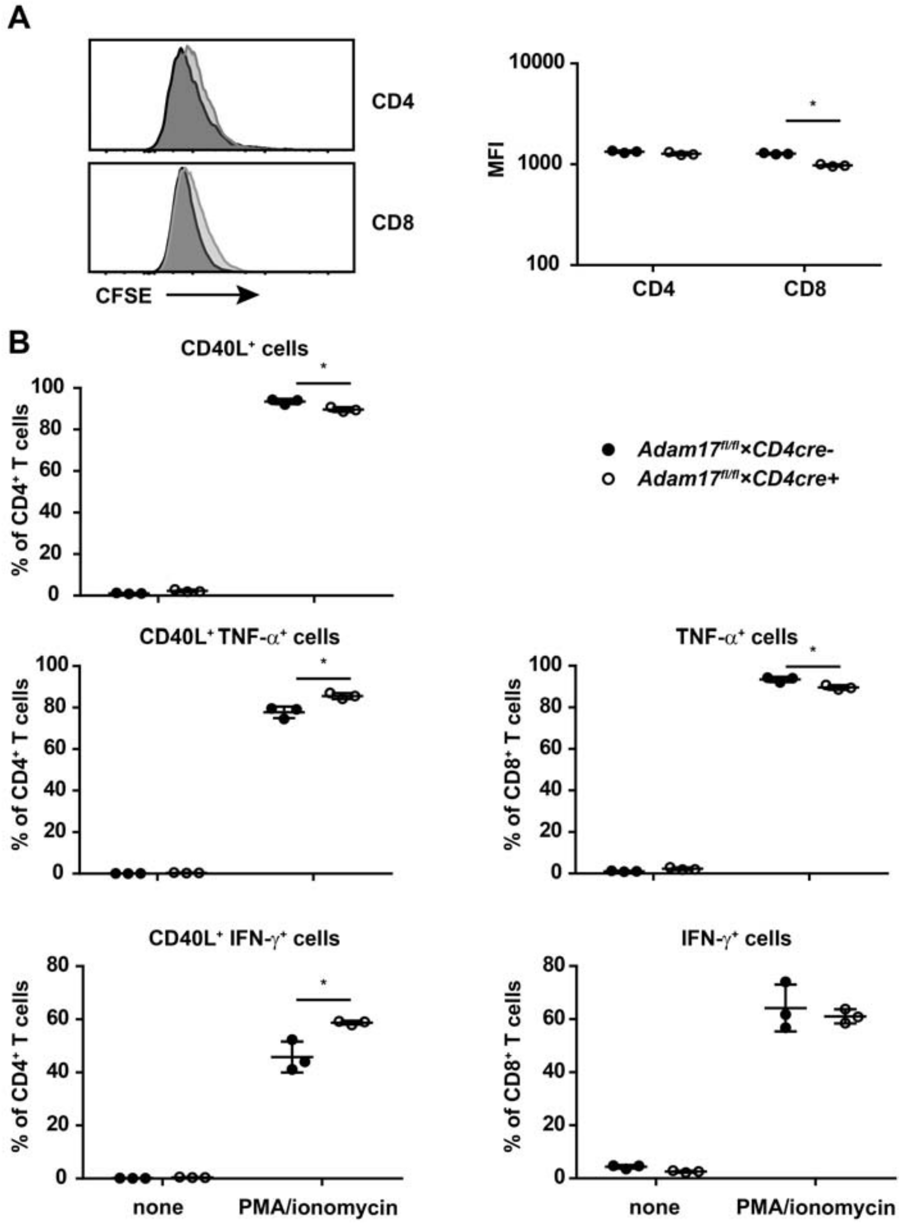

S2 Fig. *In vitro* response of T cells from *Adam17*$^{fl/fl}$×*CD4cre*$^{+}$ mice.

Spleen cells from *Adam17*$^{fl/fl}$×*CD4cre*$^{-}$ and *Adam17*$^{fl/fl}$×*CD4cre*$^{+}$ mice were labelled with CFSE and stimulated with anti-CD3 mAb and anti-CD28 mAb. After 3 days, CFSE expression was determined on CD4^{+} and CD8^{+} T cells. At this time point, T cells were also re-stimulated for further 4h with PMA and ionomycin or were left without stimulation (none). Subsequently, expression of CD40L, TNF-α and IFN-γ was determined by intracellular staining and flow cytometry. (A) Representative histograms for CFSE expression of CD4^{+} and CD8^{+} T cells from *Adam17*$^{fl/fl}$×*CD4cre*$^{-}$ (light grey) and *Adam17*$^{fl/fl}$×*CD4cre*$^{+}$ mice (dark grey). Charts give the MFI for individually analyzed samples. (B) Frequencies of CD40L^{+}, CD40L^{+}TNF-α^{+} and CD40L^{+}IFN-γ^{+} CD4^{+} T cells (left), as well as of TNF-α^{+} and IFN-γ^{+} CD8^{+} T cells (right). Charts give the mean+/-SD of triplicate cultures for each mouse strain. Groups were compared with student's t test. A p-value of <0.05 was considered significant. Data are representative for two independent experiments with similar outcome.
