# Supplementary material for: The role of ADAM17 in the T-cell response against bacterial pathogens
Source: PLoS One. 2017 Sep 6;12(9):e0184320. doi: 10.1371/journal.pone.0184320 (PMC5587322; doi:10.1371/journal.pone.0184320)
Supplement: S3 Fig — Adam17fl/fl×CD4cre- and Adam17fl/fl×CD4cre+ mice were infected with 2×10^4^ LmOVA. Spleen cells from naive mice (A) and mice infected for 8 (B) and 15 days (C) were analyzed for surface expression by flow cytometry. Scatter plots give MFI (mean fluorescence intensity) for CD62L and IL-6Rα on CD44+CD4+ and CD44+CD8+ T cells. Results for individually analyzed mice and mean +/- SD are presented. Experiments were repeated 2 times. (PDF) [file pone.0184320.s003.pdf]

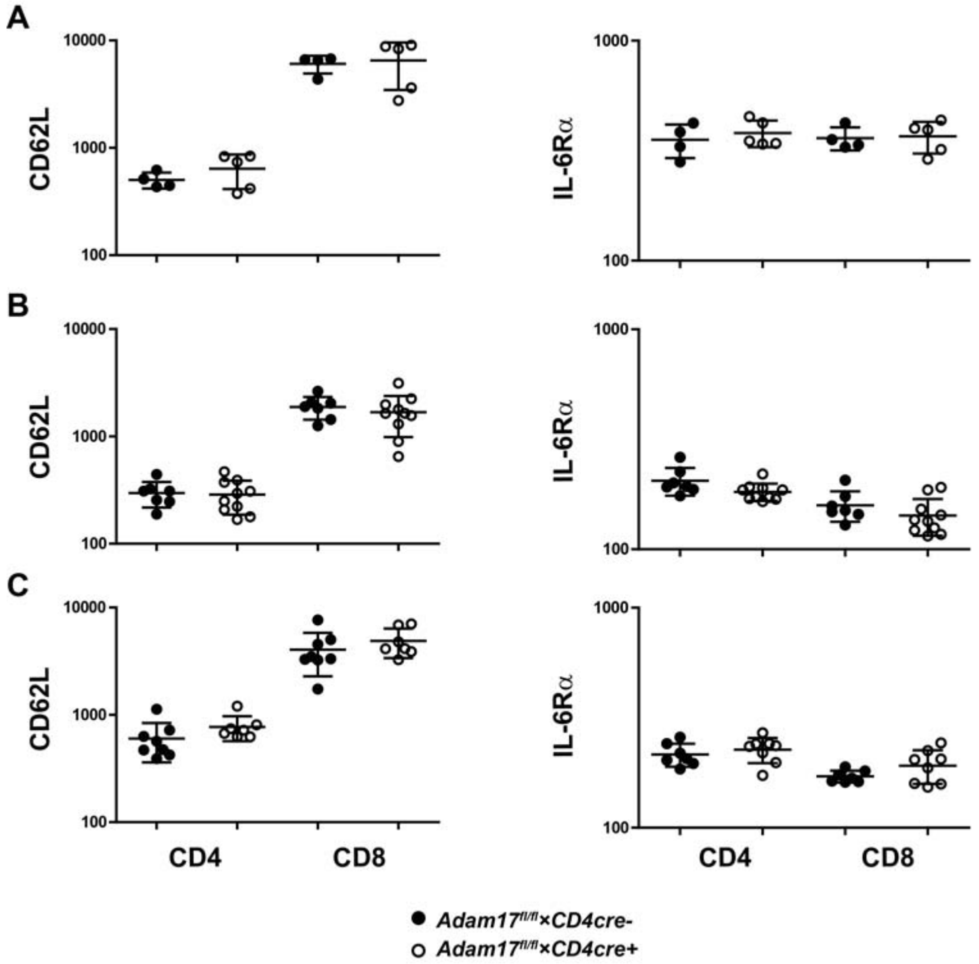

S3 Fig. CD62L and IL-6Rα surface expression on CD4^{+} and CD8^{+} T cells from *Adam17*$^{fl/fl}$×*CD4cre*$^{+}$ mice.

Adam17$^{fl/fl}$×*CD4cre*$^{-}$ and *Adam17*$^{fl/fl}$×*CD4cre*$^{+}$ mice were infected with 2×10^4 LmOVA. Spleen cells from naive mice (A) and mice infected for 8 (B) and 15 days (C) were analyzed for surface expression by flow cytometry. Scatter plots give MFI (mean fluorescence intensity) for CD62L and IL-6Rα on CD44^{+}CD4^{+} and CD44^{+}CD8^{+} T cells. Results for individually analyzed mice and mean +/- SD are presented. Experiments were repeated 2 times.
